# The Personality Inventory for DSM-5 Short Form (PID-5-SF): psychometric properties and association with big five traits and pathological beliefs in a Norwegian population

**DOI:** 10.1186/s40359-016-0169-5

**Published:** 2016-12-07

**Authors:** Jens C. Thimm, Stian Jordan, Bo Bach

**Affiliations:** 1 Department of Psychology, University of Tromsø, 9037 Tromsø, Norway; 2 Sámi Norwegian National Advisory Unit on Mental Health and Substance Use, Finnmark Hospital Trust, Karasjok, Norway; 3 Centre of Excellence on Personality Disorder, Region Zealand, Denmark

**Keywords:** PID-5, DSM-5 Section III, Personality disorders, Personality traits, Personality beliefs, Five-factor model

## Abstract

**Background:**

With the publication of the fifth edition of the *Diagnostic and Statistical Manual of Mental Disorders* (DSM-5), an alternative model for personality disorders based on personality dysfunction and pathological personality traits was introduced. The Personality Inventory for DSM-5 (PID-5) is a 220-item self-report inventory designed to assess the personality traits of this model. Recently, a short 100-item version of the PID-5 (PID-5-SF) has been developed. The aim of this study was to investigate the score reliability and structure of the Norwegian PID-5-SF. Further, criterion validity with the five factor model of personality (FFM) and pathological personality beliefs was examined.

**Methods:**

A derivation sample of university students (*N* = 503) completed the PID-5, the Big Five Inventory (BFI), and the Personality Beliefs Questionnaire – Short Form (PBQ-SF), whereas a replication sample of 127 students completed the PID-5-SF along with the aforementioned measures.

**Results:**

The short PID-5 showed overall good score reliability and structural validity. The associations with FFM traits and pathological personality beliefs were conceptually coherent and similar for the two forms of the PID-5.

**Conclusions:**

The results suggest that the Norwegian PID-5 short form is a reliable and efficient measure of the trait criterion of the alternative model for personality disorders in DSM-5.

**Electronic supplementary material:**

The online version of this article (doi:10.1186/s40359-016-0169-5) contains supplementary material, which is available to authorized users.

## Background

In the revision of the fifth edition of the *Diagnostic and Statistical Manual of Mental Disorders* (DSM-5; [3]), the DSM-5 Personality and Personality Disorders Workgroup developed a model for the diagnosis of personality disorders (PD) based on a dimensional conceptualization to address the criticisms against the categorical approach to personality disorders of the DSM-IV-TR [2]. Some of the well-acknowledged problems of the DSM-IV-TR approach are high comorbidity across PD diagnoses, inadequate coverage of personality pathology, arbitrary thresholds, temporal instability, heterogeneity within categories, and a weak scientific base of most categories (for reviews see [37, 53]). However, the Scientific Review Committee of the DSM-5 refused to adopt the proposed PD model, but it was decided to include it in Section III as “Alternative DSM-5 Model for Personality Disorders” (DSM-5 AMPD) for further investigation while the categorical approach of DSM-IV-TR [2] was retained verbatim in DSM-5 Section II (for an account of the revision process see [58]).

According to the DSM-5 AMPD, PDs are characterized by impairment of personality functioning (Criterion A) and the presence of pathological personality traits (Criterion B). Additionally, the alternative DSM-5 model offers an opportunity to diagnose six retained PD types (Antisocial, Avoidant, Borderline, Narcissistic, Obsessive-compulsive, and Schizotypal PD) conceptualized as combinations of impairments in specific domains of personality functioning and personality traits. Criterion B of the DSM-5 AMPD comprises 25 pathological personality trait facets that are organized into five broad higher order trait domains (i.e., Negative affectivity, Detachment, Psychoticism, Antagonism, and Disinhibition) [3]. For a detailed description of the personality trait facets and domains of the DSM-5 AMPD, we refer to Section III of the DSM-5 [3] and to Krueger and Markon [31]. A similar model for the diagnosis of PDs based on the assessment of the severity of personality disturbance and five traits domain is proposed for the 11^th^ revision of the International Classification of Diseases, which is due by 2018 [49].

The Personality Inventory for DSM-5 (PID-5; [4]) is a self-report inventory that was developed simultaneously with the DSM-5 AMPD pathological personality trait taxonomy to aid the assessment of these traits. The PID-5 is the result of three waves of data collection in which 37 maladaptive personality traits were reduced to 25 traits to be included in the instrument [30]. These traits are measured with 220 items. In addition, a brief 25-item form measuring only the five trait domains [5] and an informant report form of the PID-5 [34] are available.

Despite the short time since its publication, the research on the psychometric properties of the PID-5 in terms of internal consistency, test-retest reliability, and validity has been extensive and reviewed by Krueger and Markon [31] and Al-Dajani, Gralnick, and Bagby [1]. The scale development study [30] and subsequent examinations showed that the internal consistency of the PID-5 trait domains and facets is acceptable. The PID-5 scale scores have further shown stability over an average of 1.44 years in a clinical sample [54]. Few et al. [21] found a high convergence between self-reported and clinician rated PID-5 traits. A number of studies have examined how the domains and facets of the five-factor model of personality (FFM) are related to the PID-5 (e.g., [18, 21, 27, 46, 55]). The results demonstrate that the PID-5 largely converges with the FFM. Concerning psychopathology, it has been shown that PID-5 traits predict symptom counts of DSM-IV/DSM-5 section II PD categories (e.g., [6, 8, 13, 56]). Further, a high degree of overlap between common mental health problems and PID-5 traits has been found (e.g., [25, 59]). It has also been demonstrated that the PID-5 traits are associated with psychosocial and functional impairment [29, 55, 59]. Finally, constructs from cognitive therapy and schema therapy that are assumed the core of personality pathology (dysfunctional beliefs, early maladaptive schemas, schema modes; [14, 57]) can be well integrated with the PID-5 model [10, 24].

The PID-5 has been translated into several languages, including Spanish [26], French [43], German [59], Danish [15], Dutch [12], and Norwegian [50]. In a previous study [47], the Norwegian version of the original 220 items PID-5 showed adequate to high internal consistency with alphas ranging from .72 (Irresponsibility) to .95 (Eccentricity) in a university student sample. An exploratory factor analysis with CF-Equamax oblique rotation confirmed five higher factors that were congruent with other international findings. Deviating from the expected pattern, though in line with previous findings, perseveration and rigid perfectionism loaded on psychoticism instead of Negative affectivity and Disinhibition, respectively. Findings further indicated measurement invariance across a matched sample of US students [47].

However, despite its established reliability and validity, the length of the PID-5 may limit its use in clinical practice and research. On the other hand, the brief form of the PID-5 assesses only the broad domains of the trait model, but does not cover the trait facets, which are particularly informative for the clinician. Using item response theory, Maples et al. [33] developed an abridged form of the PID-5 with a smaller set of items (four items per scale). The shortened PID-5 (hereafter referred to as PID-5-SF) showed adequate internal consistency with alpha coefficients ranging from .89 to .91 (trait domains) and .74 to .88 (trait facets) with means of .90 and .83, respectively. The factor structure of the PID-5-SF was highly similar to the original form (congruency coefficients from .93 to .99). The convergent correlations ranged for the domains from .96 to .98 (mean .97) and from .89 to 1.0 (mean .94) for the facets. The similarity of the discriminant validity of the original and shortened PID-5 (the pattern of the correlations of a given domain with the four other domains) was .98. Finally, the criterion validity with the FFM, interviewer-rated Section II and Section III scores, and internalizing and externalizing outcomes was nearly identical for both forms of the PID-5. These findings suggest that the DSM-5 AMPD traits can be reliably and validly measured with a reduced set of PID-5 items without loss of information [33]. Recently, comparing all three forms of the PID-5, [10] largely replicated these findings for the Danish version of the PID-5. The Danish PID-5-SF showed satisfactory reliability and structural validity as well as a high profile agreement with the original form regarding correlations with interviewer-rated DSM-5 Section II PD symptom counts. In addition, all three forms discriminated between psychiatric patients and community-dwelling adults [9].

Extending previous research on the original PID-5 in Norway, the present study aimed to investigate the psychometric properties of the Norwegian PID-5-SF by examining the score reliability of its scales, its factor structure (structural validity), as well as the associations with normal FFM traits and core beliefs associated with the DSM-IV/DSM-5 PD categories (criterion validity).

## Method

### Participants and procedure

This study used the same sample as the previous investigation on the Norwegian PID-5 [47] comprising students from a large Norwegian university, invited by email to participate in the study. The sample consisted of 503 participants (76% female) with a mean age of 25.4 years (SD = 6.9, range 18 to 66 years). In addition, a replication sample comprising 127 students (mean age = 27.5 years, SD = 8.8, range 19 to 67 years; 65% female) was recruited for the present investigation in order to test psychometric features of the PID-5-SF as a standalone measure.

### Measures

The *Personality Inventory for DSM-5* (PID-5; [4]) is a 220-item self-report inventory designed to assess the 25 pathological personality trait facets and the five higher-order domains of the criterion B of the DSM-5 AMPD. The 25 scales are comprised of four (Submissiveness) to 14 items (Callousness, Depressiveness, and Risk taking). Items are rated on a four-point Likert scale from 0 (very false or often false) to 3 (very true or often true). In the present study, the 100 items of the PID-5-SF (four items per scale) were extracted from the original PID-5 by means of the scoring algorithm provided by Maples et al. [33]. Domain scores of the original PID-5 and the PID-5-SF were calculated by adding scores of the three scales that contribute primarily to the respective domain, i.e., Emotional lability, Anxiousness, Separation insecurity (Negative affectivity), Withdrawal, Anhedonia, Intimacy avoidance (Detachment), Unusual beliefs and experiences, Eccentricity, Perceptual dysregulation (Psychoticism), Manipulativeness, Deceitfulness, Grandiosity (Antagonism), and Irresponsibility, Impulsivity, Distractibility (Disinhibition) [4]. As the associations between the original PID-5 and the PID-5-SF and the similarity of correlations of the two forms with external variables are likely to be inflated when the PID-5-SF scales are derived from the original PID-5 (cf. [45]), the replication sample completed the PID-5-SF as a standalone measure.

The *Big Five Inventory* (BFI; [20, 28]) assesses the personality dimensions of Neuroticism, Extraversion, Openness, Agreeableness, and Conscientiousness with 44 items, scored on a five-point Likert scale ranging from 1 (disagree strongly) to 5 (agree strongly). In the present study, the Cronbach’s alphas for the five scales ranged from .73 (Agreeableness) to .86 (Extraversion).

The *Personality Beliefs Questionnaire – Short Form* (PBQ-SF; [16]) is a 65-item self-report inventory designed to assess dysfunctional cognitions associated with the DSM-IV/DSM-5 PD categories. The response items are scored on a five-point Likert scale ranging from 0 (I don’t believe it at all) to 4 (I believe it totally). In the present study, the Cronbach’s alphas for the scales ranged from .75 (antisocial and narcissistic beliefs) to .91 (paranoid beliefs). The PBQ-SF was translated into Norwegian by the first author with permission by A. T. Beck and back-translated by a professional translator unfamiliar with the English version. Discrepancies between the back-translation and the original were discussed until consensus on the Norwegian translation was reached.

### Data analytic procedures

A series of confirmatory factor analyses (CFA) was conducted to test the unidimensionality of the PID-5-SF scales. The PID-5-SF items were treated as ordinal variables, and the robust weighted least squares (WLSMW) estimator was used. Model fit was evaluated using the comparative fit index (CFI). The reliability of the Norwegian PID-5-SF was examined by calculating the internal consistencies of the facet and domain scores (Cronbach’s alpha), mean inter-item correlations, and item-total correlations. According to Clark and Watson [17], mean inter-item correlations should generally fall between .15 and .50. In order to inspect item-discrimination for each scale, we estimated and averaged their item-total correlations. To investigate the factor structure of the Norwegian PID-5-SF, an exploratory factor analysis (EFA) with CF-Equamax oblique rotation was performed using robust maximum likelihood estimator. Congruency coefficients with the factor loadings obtained in the study on the Norwegian version of the original PID-5 [47], with the loading matrix of the original PID-5 in the construction study by Krueger et al. [30], and the loading matrix of the PID-5-SF presented by Maples et al. [33] were computed. The relationships of the original and the short PID-5 with the BFI and the PBQ-SF were explored using correlation analyses. Double entry intraclass correlation coefficients (ICC; [35]) were calculated to examine the profile agreement between the original PID-5 and the PID-5-SF across the associations with the FFM and dysfunctional beliefs.

The confirmatory and exploratory factor analyses were conducted in MPlus 7.03 [40]. Factor congruence coefficients and Fisher’s r to z and z to r transformations to calculate mean correlations were computed with the psych package for R [42]. SPSS 23.0 was used for the remaining analyses.

## Results

### Derivation study using PID-5-SF data extracted from the original PID-5

In the derivation sample, alpha coefficients for the PID-5-SF domain scores ranged from .85 (Antagonism) to .98 (Negative affectivity) and for the facet scores from .60 (Perceptual dysregulation) to .90 (Depressivity). The mean alpha was .87 for the domain scores and .80 for the facet scores. The mean inter-item correlations for the PID-5-SF ranged from .32 (Antagonism) to .39 (Negative affectivity) for the domains, and from .28 (Irresponsibility) to .70 (Depressivity) for the facets with an average of .35 (domains) and .51 (facets), respectively. With regard to mean item-total correlations, the values for the domains ranged from .52 (Antagonism) to .59 (Negative affectivity), and for the facets from .39 (Irresponsibility) to .84 (Attention seeking) with an average of .55 (domains) and .63 (facets), respectively. As shown in Table 1, the CFI ranged from .98 to 1.00 for the PID-5-SF scales, indicating good model fits and unidimensionality.

**Table 1:** Factor loadings, item-level CFA, alpha coefficients, mean item-total correlations, and mean inter-item correlations of the PID-5-SF scales

PID-5-SF scales	NE		DE		PS		AN		DI		CFI		α		MII		MIT	
	D	S	D	S	D	S	D	S	D	S	D	S	D	S	D	S	D	S
Negative affectivity													.89	.89	.39	.39	.59	.59
Anxiousness*	**.68**	**.71**	.11	.18	.14	.18	.03	−.07	.04	−.05	.99	1.00	.84	.82	.57	.53	.67	.65
Emotional lability*	**.68**	**.55**	−.08	.12	.18	.16	−.05	−.21	.16	.31	.95	.96	.81	.82	.53	.53	.64	.65
Hostility	**.51**	**.49**	.04	.03	.10	.00	.21	.05	.08	.19	.99	.98	.80	.79	.49	.46	.65	.64
Perseveration	.27	.23	.20	.31	.25	.16	−.04	.16	.36	.33	.99	.99	.77	.76	.47	.46	.59	.59
Restricted affectivity	−.32	−.33	**.62**	**.45**	.15	.21	.22	.31	.10	−.02	.98	1.00	.80	.77	.50	.45	.61	.58
Seperation insecurity*	**.55**	**.61**	.00	.27	.01	−.16	.01	.09	.11	.09	.99	1.00	.81	.80	.51	.49	.63	.62
Submissiveness	.32	.32	.23	.18	−.12	.18	.10	−.13	.22	.20	1.00	1.00	.81	.79	.51	.48	.62	.60
Detachment													.87	.88	.37	.38	.57	.51
Anhedonia*	.33	.17	**.62**	**.71**	.03	.03	−.04	.22	.22	.10	1.00	1.00	.76	.77	.46	.49	.58	.60
Depressivity	.29	.07	**.60**	**.81**	.09	.08	−.10	−.02	.17	.05	1.00	.95	.90	.87	.70	.64	.78	.75
Intimacy avoidance*	−.11	−.16	.39	.40	.29	**.46**	.10	.04	.04	−.15	1.00	1.00	.86	.86	.60	.60	.73	.71
Suspiciousness	.26	**.47**	.29	.14	.32	.30	.25	.15	.04	.02	1.00	1.00	.68	.69	.36	.38	.47	.49
Withdrawal*	.07	.01	**.67**	.37	.14	**.46**	.14	.23	−.03	−.25	1.00	1.00	.83	.84	.56	.57	.67	.69
Psychoticism													.86	.86	.33	.33	.54	.54
Eccentricity*	−.02	.03	.23	.04	**.51**	**.76**	.02	.16	.25	.08	1.00	1.00	.88	.84	.65	.58	.74	.68
Perceptual dysregulation*	.07	.05	−.06	−.04	**.70**	**.54**	.03	.06	.03	.18	.99	.96	.60	.66	.29	.32	.40	.45
Unusual beliefs and exp.*	.00	.12	−.15	−.18	**.78**	**.60**	−.03	−.16	.03	.11	.99	1.00	.72	.78	.40	.47	.52	.61
Antagonism													.85	.85	.32	.32	.52	.53
Attention seeking	.07	.22	**−.41**	−.33	.00	−.07	.40	.16	.23	**.45**	.99	1.00	.88	.88	.66	.66	.84	.76
Callousness	−.14	−.11	.31	.11	.15	−.03	**.58**	**.84**	−.07	−.11	1.00	1.00	.83	.80	.59	.51	.68	.63
Deceitfulness*	.07	.28	.05	−.09	.03	.09	**.66**	**.48**	.22	.30	1.00	1.00	.69	.71	.36	.39	.48	.48
Grandiosity*	.00	.00	.01	−.12	.16	.15	**.63**	**.64**	−.11	.10	1.00	.99	.76	.75	.44	.43	.56	.56
Manipulativeness*	.06	.27	−.13	−.17	.01	.04	**.70**	.40	.14	.37	.99	1.00	.77	.76	.45	.45	.57	.57
Disinhibition													.86	.87	.33	.35	.54	.56
Distractibility*	.19	.10	.18	.39	.11	−.01	−.12	.12	**.56**	**.53**	1.00	1.00	.88	.90	.64	.69	.74	.77
Impulsivity*	−.05	−.13	−.12	.10	.06	.08	.03	−.01	**.76**	**.77**	1.00	1.00	.79	.74	.49	.42	.61	.54
Irresponsibility*	.01	.08	.08	.28	.09	.13	.26	.12	**.48**	**.47**	1.00	1.00	.61	.59	.28	.27	.39	.38
Rigid perfectionism	.32	**.67**	.20	−.16	.20	.23	.14	.13	−.02	.00	1.00	1.00	.83	.74	.55	.41	.67	.55
Risk taking	−.31	−.09	−.05	−.14	.17	.28	.20	.18	**.45**	**.60**	1.00	.99	.80	.81	.50	.52	.62	.63
Factor congruence with the Norwegian original form	.98	.88	.97	.86	.92	.87	.98	.80	.94	.88								
Factor congruence with Krueger et al.’s [30] original form	.95	.87	.93	.79	.95	.90	.93	.77	.76	.66								
Factor congruence with Maples et al.’s [33] shortened form	.89	.85	.86	.75	.89	.80	.90	.79	.89	.87								

*D* derived short form; *S* standalone short form. Factor loadings above .40 are in bold. EFA with Oblique CF-Equamax rotation was used. * PID-5 scales used to compute domain scores. *NE* negative affectivity, *DE* detachment, *PS* psychoticism, *AN* antagonism, *DI* disinhibition. *CFI* comparative fit index; *α* Cronbach’s alpha coefficient, *MII* mean inter-item correlations, *MIT* mean item-total correlations

The results from EFA with CF-Equamax oblique rotation of the PID-5-SF are also displayed in Table 1. The model fit the data reasonably well (*χ* ^2^ = 543.83, *p* < .001, df = 185; RMSEA = .06, CFI = .92, SRMR = .03). The factor loadings showed largely the expected pattern. Deviating from the proposed factor structure of the PID-5, Perseveration, assumed to belong to the Negative affectivity domain, had its highest loading on Disinhibition. Suspiciousness (Detachment or Negative affectivity) had its strongest loading on Psychoticism, Attention seeking (Antagonism) negatively on Detachment, and Rigid perfectionism (Disinhibition) on Negative affectivity. Congruence coefficients of the factors of the original PID-5 and the PID-5-SF ranged from .92 (Psychoticism) to .98 (Negative affectivity and Antagonism) with a mean of .96. Factor congruence with the loadings matrix reported by Krueger et al. [30] for the original PID-5 ranged from .76 (Disinhibition) to .95 (Negative affectivity and Psychoticism). Congruency coefficients with the loadings presented by Maples et al. [33] for the PID-5-SF ranged from .86 (Detachment) to .90 (Antagonism).

To explore the relationships between the PID-5-SF and the FFM and personality beliefs, PID-5 traits were correlated with the BFI and PBQ-SF scales. Associations between the PID-5 domains and the BFI scales are presented in Table 2. Negative affectivity was highly correlated with Neuroticism, Detachment (negatively) with Extraversion, Antagonism (negatively) with Agreeableness, and Disinhibition (negatively) with Conscientiousness. Psychoticism was moderately correlated with all BFI scales. Double entry ICCs indicated almost perfect profile agreement between the domains of the two forms of the PID-5 (ranging from .99 to 1.00).

**Table 2:** Correlations between PID-5 domains and BFI scales

	BFI scales															R^2^			Profile agreement	
	Neuroticism			Extraversion			Openness			Agreeableness			Conscientious-ness							
PID-5-SF domains	**O**	**D**	**S**	**O**	**D**	**S**	**O**	**D**	**S**	**O**	**D**	**S**	**O**	**D**	**S**	**O**	**D**	**S**	**O-D**	**O-S**
Negative affectivity	**.77**	**.73**	**.76**	**-.24**	**−.22**	−.10	**−.10**	**−.11**	.02	**−.23**	**−.21**	**−.28**	**−.31**	**−.29**	**−.25**	.60	.54	.59	1.00	.97
Detachment	**.44**	**.42**	**.41**	**−.69**	**−.61**	**−.54**	**−.10**	−.07	.15	**−.52**	**−.48**	**−.39**	**−.36**	**−.35**	**−.40**	.59	.49	.49	.99	.93
Psychoticism	**.35**	**.27**	**.32**	**−.33**	**−.29**	**−.26**	**.26**	**.25**	**.35**	**−.43**	**−.39**	**−.24**	**−.41**	**−.33**	**−.29**	.40	.31	.29	.99	.94
Antagonism	.01	.02	**.21**	−.05	−.07	.10	**.19**	**.15**	**.21**	**−.48**	**−.48**	**−.36**	**−.19**	**−.18**	**−.36**	.29	.28	.30	1.00	.83
Disinhibition	**.40**	**.38**	**.34**	**−.23**	**−.19**	.01	.08	**.11**	**.19**	**−.36**	**−.34**	**−.33**	**−.72**	**−.69**	**−.78**	.57	.53	.69	1.00	.95

R^2^ indicates the degree to which all BFI scales account for each PID-5 domain score (all *p*s < .001). *O* original form; *D* derived short form; *S* standalone short form. Coefficients in bold are significant at *p* < 0.05

The results of the correlations of the domain and facet scores of the original and the short PID-5 with the PBQ-SF scales are shown in Table 3. Results indicate that each PBQ-SF had several significant associations with the scales of the original and shortened PID-5. The mean profile agreement between the original PID-5 and PID-5-SF across the PBQ-SF scales was .99 (domains) and .96 (facets) with ranges from .82 (Psychoticism) to 1.00 (Negative affectivity, Detachment, Antagonism) for the domains and -.30 (Perceptual dysregulation) to 1.00 (Separation insecurity) for the facets. (The beta weights from the regression analyses predicting PID-5 and PID-5-SF trait domains and facets from the BFI and PBQ-SF scales, respectively, are included in the online Additional file 1).

**Table 3:** Correlations of PID-5 scales with Personality Beliefs scales

PID-5 scales	PAR			SCD			ANT			BDL			HIS			NAR			AVD			DPT			OBS			PAG			R^2^			Profile agreement	
	O	D	S	O	D	S	O	D	S	O	D	S	O	D	S	O	D	S	O	D	S	O	D	S	O	D	S	O	D	S	O	D	S	O-D	O-S
Negative affectivity	**.43**	**.43**	**.50**	**.14**	**.13**	**.24**	**.28**	**.28**	**.36**	**.73**	**.71**	**.74**	**.51**	**.52**	**.57**	**.17**	**.20**	**.34**	**.64**	**.62**	**.68**	**.73**	**.73**	**.76**	**.46**	**.45**	**.56**	**.29**	**.29**	**.43**	.62	.61	.66	1.00	.88
Anxiousness	**.47**	**.44**	**.44**	**.25**	**.22**	**.27**	**.30**	**.28**	**.27**	**.68**	**.63**	**.62**	**.44**	**.41**	**.39**	**.15**	**.16**	**.25**	**.61**	**.56**	**.57**	**.61**	**.57**	**.60**	**.47**	**.44**	**.52**	**.30**	**.27**	**.39**	.50	.43	.48	.98	.93
Emotional lability	**.32**	**.35**	**.45**	**.12**	**.15**	**.28**	**.17**	**.21**	**.34**	**.59**	**.60**	**.65**	**.41**	**.44**	**.50**	**.13**	**.16**	**.36**	**.53**	**.54**	**.59**	**.56**	**.57**	**.65**	**.32**	**.33**	**.49**	**.25**	**.29**	**.45**	.41	.41	.50	.99	.61
Perseveration	**.45**	**.41**	**.43**	**.43**	**.37**	**.42**	**.38**	**.34**	**.41**	**.56**	**.56**	**.60**	**.41**	**.40**	**.42**	**.24**	**.21**	**.40**	**.52**	**.52**	**.58**	**.48**	**.48**	**.54**	**.53**	**.41**	**.43**	**.48**	**.44**	**.55**	.44	.38	.47	.86	.62
Hostility	**.46**	**.33**	**.28**	**.42**	**.29**	.14	**.53**	**.37**	**.34**	**.47**	**.45**	**.40**	**.41**	**.36**	**.31**	**.35**	**.22**	**.29**	**.49**	**.46**	**.40**	**.39**	**.41**	**.44**	**.36**	**.33**	**.44**	**.51**	**.34**	**.42**	.40	.28	.32	.06	-.19
Restricted affectivity	**.45**	**.43**	**.23**	**.54**	**.51**	**.37**	**.41**	**.36**	**.22**	**.33**	**.31**	**.27**	**.19**	**.17**	.08	**.21**	**.18**	.05	**.34**	**.32**	**.24**	**.21**	**.19**	.11	**.30**	**.27**	.02	**.44**	**.40**	**.20**	.36	.31	.25	.97	.16
Separation insecurity	**.27**	**.27**	**.35**	-.07	-.07	.06	**.21**	**.21**	**.30**	**.55**	**.53**	**.59**	**.46**	**.44**	**.53**	**.15**	**.17**	**.25**	**.45**	**.43**	**.55**	**.68**	**.66**	**.67**	**.34**	**.33**	**.39**	**.15**	**.15**	**.23**	.53	.50	.50	1.00	.92
Submissiveness	**.21**	**.21**	**.32**	**.09**	**.09**	.15	**.22**	**.22**	.13	**.40**	**.40**	**.45**	**.36**	**.36**	**.44**	.06	.06	.13	**.40**	**.40**	**.40**	**.46**	**.46**	**.41**	**.32**	**.32**	**.33**	**.12**	**.12**	**.20**	.28	.28	.31	1.00	.86
Detachment	**.56**	**.55**	**.51**	**.67**	**.65**	**.71**	**.40**	**.40**	**.30**	**.64**	**.63**	**.56**	**.28**	**.27**	.15	**.17**	**.17**	**.20**	**.64**	**.62**	**.56**	**.44**	**.43**	**.39**	**.45**	**.45**	**.25**	**.49**	**.47**	**.44**	.66	.63	.70	1.00	.83
Anhedonia	**.46**	**.47**	**.41**	**.38**	**.35**	**.43**	**.30**	**.33**	**.32**	**.67**	**.68**	**.69**	**.33**	**.35**	**.41**	**.11**	**.12**	**.22**	**.60**	**.59**	**.58**	**.54**	**.56**	**.58**	**.36**	**.40**	**.30**	**.39**	**.38**	**.38**	.50	.49	.56	.99	.93
Depressivity	**.52**	**.45**	**.28**	**.36**	**.33**	**.33**	**.32**	**.29**	**.21**	**.78**	**.72**	**.69**	**.42**	**.32**	**.38**	**.08**	.07	.05	**.67**	**.60**	**.53**	**.63**	**.55**	**.55**	**.45**	**.39**	**.26**	**.39**	**.34**	**.23**	.65	.56	.62	.95	.77
Intimacy avoidance	**.40**	**.37**	**.32**	**.60**	**.59**	**.61**	**.31**	**.30**	.14	**.35**	**.33**	**.26**	**.14**	**.12**	-.11	**.13**	**.11**	.07	**.41**	**.38**	**.29**	**.20**	**.17**	.13	**.30**	**.28**	.09	**.34**	**.31**	**.26**	.40	.39	.50	.99	.68
Suspiciousness	**.77**	**.75**	**.67**	**.46**	**.47**	**.31**	**.54**	**.56**	**.44**	**.64**	**.65**	**.62**	**.38**	**.43**	.**37**	**.28**	**.35**	**.37**	**.55**	**.55**	**.45**	**.47**	**.48**	**.51**	**.39**	**.41**	**.49**	**.49**	**.51**	**.53**	.63	.61	.55	.97	.74
Withdrawal	**.52**	**.47**	**.49**	**.67**	**.62**	**.65**	**.38**	**.34**	**.26**	**.56**	**.52**	**.42**	**.23**	**.19**	.07	**.18**	**.18**	**.20**	**.58**	**.53**	**.48**	**.36**	**.32**	**.25**	**.44**	**.41**	**.22**	**.48**	**.45**	**.41**	.60	.51	.56	.96	.74
Psychoticism	**.59**	**.54**	**.44**	**.51**	**.49**	**.56**	**.44**	**.42**	**.34**	**.55**	**.47**	**.43**	**.38**	**.31**	**.28**	**.32**	**.32**	**.36**	**.48**	**.41**	**.36**	**.43**	**.35**	**.34**	**.39**	**.34**	**.38**	**.57**	**.54**	**.52**	.47	.40	.44	.82	.46
Eccentricity	**.55**	**.53**	**.47**	**.49**	**.49**	**.63**	**.37**	**.36**	**.42**	**.53**	**.49**	**.45**	**.34**	**.30**	**.25**	**.27**	**.27**	**.37**	**.45**	**.42**	**.43**	**.39**	**.35**	**.36**	**.36**	**.34**	**.35**	**.54**	**.53**	**.54**	.43	.41	.48	.96	.70
Perceptual dysregulation	**.52**	**.35**	**.32**	**.43**	**.31**	**.36**	**.43**	**.37**	**.23**	**.56**	**.31**	**.34**	**.39**	**.22**	**.25**	**.25**	**.28**	**.21**	**.50**	**.27**	**.25**	**.46**	**.25**	**.30**	**.38**	**.23**	**.23**	**.49**	**.40**	**.34**	.41	.21	.23	-.30	-.28
Unusual beliefs	**.44**	**.36**	**.24**	**.37**	**.32**	**.30**	**.38**	**.30**	.14	**.31**	**.28**	**.22**	**.23**	**.23**	**.20**	**.35**	**.25**	**.25**	**.25**	**.26**	.15	**.23**	**.21**	.15	**.26**	**.22**	**.31**	**.44**	**.35**	**.34**	.27	.17	.25	.62	.01
Antagonism	**.43**	**.40**	**.51**	**.42**	**.42**	**.38**	**.63**	**.62**	**.63**	**.25**	**.24**	**.48**	**.42**	**.39**	**.54**	**.55**	**.57**	**.68**	**.26**	**.25**	**.40**	**.17**	**.16**	**.43**	**.27**	**.27**	**.40**	**.51**	**.52**	**.55**	.49	.49	.60	1.00	.44
Attention seeking	**.22**	**.13**	.11	**.14**	.05	.05	**.35**	**.27**	**.29**	**.12**	.05	.17	**.50**	**.46**	**.48**	**.42**	**.38**	**.40**	.07	.00	.09	**.17**	**.13**	**.22**	**.15**	**.10**	**.25**	**.29**	**.19**	.17	.37	.34	.37	.87	.85
Callousness	**.47**	**.38**	**.44**	**.54**	**.47**	**.36**	**.61**	**.53**	**.47**	**.27**	**.23**	**.26**	**.16**	**.12**	.10	**.45**	**.40**	**.41**	**.25**	**.22**	**.21**	**.12**	**.12**	.16	**.19**	**.15**	.11	**.56**	**.47**	**.36**	.55	.42	.35	.93	.79
Deceitfulness	**.45**	**.41**	**.47**	**.40**	**.38**	**.31**	**.58**	**.57**	**.56**	**.33**	**.31**	**.49**	**.42**	**.40**	**.52**	**.37**	**.39**	**.51**	**.34**	**.31**	**.38**	**.22**	**.20**	**.46**	**.26**	**.25**	**.33**	**.47**	**.45**	**.43**	.40	.38	.45	.97	.28
Grandiosity	**.28**	**.28**	**.49**	**.32**	**.34**	**.39**	**.44**	**.47**	**.53**	**.10**	**.14**	**.31**	**.24**	**.25**	**.24**	**.64**	**.63**	**.76**	.07	**.13**	**.30**	.09	**.12**	**.26**	**.18**	**.18**	**.26**	**.41**	**.44**	**.57**	.45	.44	.64	.99	.66
Manipulativeness	**.29**	**.29**	**.33**	**.29**	**.32**	**.25**	**.49**	**.49**	**.47**	**.10**	**.14**	**.38**	**.34**	**.32**	**.52**	**.42**	**.41**	**.45**	**.15**	**.18**	**.30**	.07	**.09**	**.34**	**.21**	**.22**	**.37**	**.38**	**.39**	**.37**	.33	.31	.38	.99	.20
Disinhibition	**.39**	**.40**	**.49**	**.34**	**.32**	**.32**	**.37**	**.38**	**.47**	**.52**	**.51**	**.61**	**.44**	**.45**	**.44**	**.19**	**.20**	**.38**	**.47**	**.45**	**.54**	**.43**	**.43**	**.54**	**.24**	**.24**	**.24**	**.47**	**.45**	**.54**	.40	.37	.51	.99	.64
Distractibility	**.35**	**.33**	**.43**	**.31**	**.27**	**.30**	**.27**	**.25**	**.42**	**.53**	**.50**	**.59**	**.39**	**.37**	**.41**	**.11**	**.12**	**.31**	**.47**	**.43**	**.54**	**.45**	**.43**	**.53**	**.28**	**.28**	**.27**	**.39**	**.36**	**.47**	.35	.30	.43	.97	.66
Impulsivity	**.26**	**.29**	**.31**	**.17**	**.18**	**.23**	**.29**	**.30**	**.34**	**.25**	**.29**	**.36**	**.34**	**.34**	**.32**	**.15**	**.16**	**.22**	**.22**	**.24**	**.36**	**.22**	**.25**	**.33**	.07	**.09**	**.18**	**.33**	**.33**	**.40**	.20	.21	.26	.96	.46
Irresponsibility	**.35**	**.33**	**.46**	**.36**	**.34**	**.24**	**.40**	**.38**	**.37**	**.43**	**.40**	**.56**	**.35**	**.36**	**.41**	**.24**	**.22**	**.37**	**.42**	**.39**	**.48**	**.33**	**.32**	**.51**	**.18**	**.17**	.17	**.46**	**.41**	**.46**	.34	.29	.46	.95	.52
Rigid perfectionism	**.40**	**.41**	**.50**	**.39**	**.35**	**.27**	**.33**	**.31**	**.40**	**.44**	**.44**	**.47**	**.30**	**.31**	**.44**	**.26**	**.23**	**.40**	**.44**	**.43**	**.50**	**.38**	**.37**	**.45**	**.76**	**.77**	**.75**	**.35**	**.33**	**.42**	.61	.60	.61	.99	.74
Risk taking	.00	**.21**	**.39**	.09	**.26**	**.30**	**.16**	**.34**	**.57**	**-.12**	**.12**	**.33**	.09	**.21**	**.27**	**.14**	**.26**	**.39**	**-.16**	**.08**	**.23**	**-.16**	.04	**.28**	-.01	**.17**	**.23**	**.21**	**.38**	**.52**	.19	.21	.40	.20	-.41

Correlations in bold are significant at *p* < .05. R^2^ indicates the degree to which all PBQ scales account for each PID-5 score (all *p*s < .001). *O* original form; *D* derived short form; *S* standalone short form. Personality Beliefs Questionnaire (PBQ-SF) scales: Paranoid (PAR), Schizoid (SCD), Antisocial (ANT), Borderline (BDL), Histrionic (HIS), Narcissistic (NAR), Avoidant (AVD), Dependent (DPT), Obsessive-Compulsive (OBS), and Passive-Aggressive (PAG)

### Replication study using the PID-5-SF as a standalone measure

In the replication sample, Cronbach’s alpha for the PID-5-SF domain scores ranged from .85 (Antagonism) to .89 (Negative affectivity) and from .59 (Irresponsibility) to .90 (Distractibility) for the facet scores. The mean alphas were .87 and .79, respectively. The mean inter-item correlations ranged from .32 (Antagonism) to .39 (Negative affectivity) for the domains, and from .27 (Irresponsibility) to .69 (Distractibility) for the facets with an average of .35 (domains) and .49 (facets), respectively. The mean item-total correlations ranged for the domains from .51 (Detachment) to .59 (Negative affectivity) and for the facets from .38 (Irresponsibility) to .77 (Distractibility) with an average of .55 (domains) and .61 (facets), respectively. The CFI ranged from .95 to 1.00, indicating good model fits and unidimensionality.

Table 1 contains the results from EFA with CF-Equamax oblique rotation of the PID-5-SF in the replication sample. The model fit was estimated (*χ* ^2^ = 365.72, *p* < .001, df = 185; RMSEA = .09, CFI = .86, SRMR = .04). The following scales had their highest loadings on other than the proposed factors: Perseveration (Negative affectivity) on Disinhibition, Intimacy avoidance and Withdrawal (Detachment) on Psychoticism, Attention seeking (Antagonism) on Disinhibition, and Rigid perfectionism (Disinhibition) on Negative affectivity. Congruence coefficients of the factors of the Norwegian PID-5 and the PID-5-SF in the replication sample ranged from .80 (Antagonism) to .88 (Negative affectivity and Disinhibition) with a mean of .86. Factor congruence with the loadings matrix reported by Krueger et al. [30] for the original PID-5 ranged from .66 (Disinhibition) to .90 (Psychoticism). Congruency coefficients with the loadings presented by Maples et al. [33] for the PID-5-SF ranged from .75 (Detachment) to .87 (Disinhibition).

Correlations between the PID-5-SF domains and the BFI in the replication sample are shown in Table 2. The profile agreement between the standalone PID-5-SF domain scores obtained in the replication sample and the PID-5-SF domain scores obtained in the derivation sample ranged from .83 (Antagonism) to .97 (Negative affectivity) with a mean of .94.

In Table 3, the correlations between the PID-5-SF and the PBQ-SF scales in the replication sample are shown. The mean profile agreement between the PID-5-SF in the replication sample and the original PID-5 in the initial sample ranged from .44 (Antagonism) to .88 (Negative affectivity) with a mean of .70 for the domains and from -.28 (Perceptual dysregulation) to .93 (Anxiousness and Anhedonia) for the facets (mean = .61).

## Discussion

It is widely recognized that the categorical approach to PDs in DSM-5 [3] has serious flaws. However, with the introduction of DSM-5, an alternative and dimensional model of PDs based on pathological personality traits and personality dysfunction is provided, which people are free to choose. The PID-5 [4] is currently the primary instrument to assess the five trait domains and 25 maladaptive personality trait facets of the DSM-5 AMPD. This 220-item inventory has shown adequate psychometric properties in clinical and nonclinical samples, in different age groups and in different countries [1]. Recently, an abbreviated form of the PID-5 with 100 items has been developed [33]. The goal of the present study was to investigate the reliability, structure, and criterion validity of the PID-5-SF in two Norwegian samples. In the first sample, the PID-5-SF was derived from the original PID-5, whereas in the second sample – the replication sample -, the PID-5-SF was used as a standalone instrument to obtain validity estimates that are not affected by biases caused by scoring the two forms from the same administration (cf. [45]).

The score reliability of the Norwegian PID-5-SF was overall good in terms of internal consistency, mean inter-item correlations, and mean item-total correlations. In the derivation sample, the mean alpha coefficients were .87 (domains) and .80 (facets), respectively. In the replication sample, the mean Cronbach’s alphas were .87 for the domains and .79 for the facets, respectively. This is remarkable given the small number of items per scale and aligns with previous findings [9, 10, 33]. However, in the present investigation, comparatively low internal consistencies were found for Perceptual dysregulation and Irresponsibility (.60 and .61 in the derivation sample and .66 and .59 in the replication sample, respectively). A similar alpha for the Irresponsibility scale of the PID-5-SF (.63) was reported by Bach et al. [9, 10].

The factor structure of the Norwegian PID-5-SF used as a standalone instrument showed similarity with the original PID-5 form. The factor congruence coefficients were .88 (Negative affectivity), .86 (Detachment), .87 (Psychoticism), .80 (Antagonism), and .88 (Disinhibition) with an average of .86. According to Lorenzo-Seva and Ten Berge [32], congruence coefficients in the range .85-.94 indicate fair similarity, and factors can be assumed equal when the values are above .95. Thus, the results suggest that the factors obtained in the analyses of the short and original Norwegian PID-5 displayed adequate similarity with the exception of Antagonism. Overall, fairly high factor congruency coefficients of the Norwegian PID-5-SF with the original PID-5 and the PID-5-SF in the US [30, 33] were found. Some scales of the PID-5-SF had their highest loadings on other factors than expected from the proposed structure of the inventory [30]. In both samples, Rigid perfectionism loaded on Negative affectivity (instead of Disinhibition) and Perseveration on Disinhibition (instead of Negative affectivity). Further, in the derivation sample, Suspiciousness loaded on Psychoticism (instead of Detachment or Negative affectivity) and Attention seeking on Detachment (instead of Antagonism). In the replication sample, Intimacy avoidance and Withdrawal loaded on Psychoticism (instead of Detachment) and Attention seeking on Disinhibition. However, these deviations have previously been observed in studies on the PID-5. Rigid perfectionism has repeatedly shown to load on Negative affectivity [12, 13, 15, 34, 43, 55]. In the Wright and Simms [55] study on the PID-5 and related measures, Perseveration loaded on Disinhibition almost as high as on Negative affectivity (.35 and .37, respectively). With regard to Suspiciousness, Bastiaens et al. [12, 13] found that this facet loaded nearly equally high on Psychoticism, Negative affectivity and Detachment. As in the present study, Attention seeking loaded about equally high on Detachment (low) and Antagonism in the investigation by Wright and Simms [55]. Substantial cross loadings of Intimacy avoidance and Withdrawal on Psychoticism have been previously reported by Maples et al. [33] and Wight and Simms [55]. Maples et al. [33] also found that Attention seeking loaded on Disinhibition.

The criterion validity of the PID-5-SF was investigated by examining the relationships with the dimensions of the FFM and dysfunctional beliefs associated with the DSM-IV/DSM-5 PD categories. Further, the similarity of these associations between the original form of the Norwegian PID-5 and the short form was examined to test if the nomological network of the original PID-5 is maintained by the short form (cf. [33]). In line with previous studies on the PID-5 and FFM (e.g., [18, 23, 55]), the PID-5 domains of the original and short form were strongly associated with the FFM dimensions in both samples: Negative affectivity with Neuroticism, (low) Detachment with Extraversion, (low) Antagonism with Agreeableness, and (low) Disinhibition with Conscientiousness. In the present study, Psychoticism was significantly related to Openness, but showed also significant associations with the remaining FFM dimensions. Findings on the relationships between Psychoticism and Openness have been mixed so far. In accordance with the results of the current study, Thomas et al. [48] and De Fruyt et al. [19] reported significant Psychoticism-Openness associations in student samples. On the other hand, several other studies have found only weak or near zero correlations between Psychoticism and Openness (e.g., [41, 51, 59]). Importantly for the purpose of the present study, when used as a standalone instrument, the profile agreement of the PID-5-SF with the original form across the FFM-dimensions was high with a mean of .94.

Further, strong conceptually meaningful associations between the PID-5 scales of the original and short form and pathological personality beliefs were found in both samples. For example, paranoid beliefs were strongly related to Suspiciousness and Schizoid beliefs to Intimacy avoidance. Antisocial beliefs predicted highly Callousness and Deceitfulness. Borderline beliefs had significant relationships with PID-5 facets from all domains, but were especially associated with Depressivity, Anxiousness, Anhedonia, Emotional lability, and Suspiciousness. Histronic beliefs were associated with Attention seeking. Narcissistic beliefs predicted primarily Grandiosity. Avoidant beliefs were most strongly related to Depressivity and Anxiousness. Dependent beliefs were primarily associated with Separation insecurity. Obsessive-compulsive beliefs were a strong predictor of Rigid perfectionism. These results are in line with the findings of Hopwood et al. [24, 25] and suggest that the cognitive perspective on PDs can be integrated with the DSM-5 section III trait model. In the replication sample, the profile agreement of the original and short form of the PID-5 was high, averaging .70 for the PID-5 domains and .61 for the PID-5 facets. It should be noted that the profile agreement was very low or even negative for several scales, including Hostility, Restricted affectivity, Perceptual dysregulation, Deceitfulness, Manipulativeness, and Risk taking.

Taken together, the findings of the present study regarding reliability, structure, and criterion validity suggest that the Norwegian PID-5 short form is a parsimonious, overall internally consistent, and structurally valid measure of the trait criterion of the DSM-5 AMPD. Fairly similar factor structures of the original PID-5 and the PID-5-SF, and, for the majority of scales, similar associations with external criteria suggest that the knowledge base that has been built around the original PID-5 can be largely applied to the shortened version. These results are in accordance with and supplement the findings of previous investigations on the PID-5-SF [9, 10, 33] and support its use in research and clinical practice. The brevity of the PID-5-SF, while retaining the comprehensiveness of the original version, makes it easier to include the pathological personality traits of the DSM-5 AMPD in clinical assessment. Widiger and Samuel [52] recommended for the assessment of the DSM-IV-TR PDs to use first a self-report inventory for screening purposes, followed by a structured interview. In a similar way, the PID-5-SF can serve as a short screening instrument used prior to an interview-based assessment, e.g., the structured interview that is currently being developed for the assessment of the traits system (criterion B) along with rating of functioning (criterion A; [22]). Although concerns regarding the clinical utility of the DSM-5 AMPD have been raised when the model was developed [58], findings support its clinical usefulness and acceptability in routine clinical practice. In a field trial of the DSM-5, the clinical utility ratings of the proposed diagnostic criteria for PDs were among the highest [39]. The pathological traits of the DSM-5 AMPD have been found to be superior to the DSM-IV-TR/DSM-5 PD categories with respect to clinicians’ ratings of ease of use, communication with patients, usefulness for describing an individual’s personality problems and global personality, and treatment planning [38]. Furthermore, the DSM-5 AMPD predicts treatment decisions (e.g., level of treatment, type of psychotherapeutic or pharmacological treatment) better than the DSM-IV-TR/DSM-5 PD categories [36]. Examples of how the DSM-5 AMPD can be used in clinical practice are provided by Skodol, Morey, Bender, and Oldham [44] and Bach, Markon, Simonsen, and Krueger [11].

A limitation of the present study is the use of a convenient nonclinical sample consisting of university students. This group is obviously rather homogeneous with respect to age, educational level, and socioeconomic status. Although the DSM-5 AMPD personality traits are assumed to be continuously distributed [3], the variance of the distribution of these traits is likely restricted in university student samples, which may affect the generalizability of the findings. Ideally, the present study is extended and replicated in more heterogeneous samples, including patients within mental health care. Another limitation of the current investigation is the relatively low sample size of the replication sample. Further, this study used only self-reported data, which may have involved a risk for artificially high correlations between measures due to shared method variance. Importantly, as few items of the original PID-5 and none of the PID-5-SF items are reversed scored and the items describe undesirable traits, these instruments are particularly prone to the effects of acquiescence responding and social desirability responding [7]. As a consequence, the alpha reliabilities and the associations with other self-report measures can be inflated [7]. It is therefore possible that the results of the present study would have been different if reports from multiple informants (e.g., spouse, parents, or siblings) had been available. More definitive findings would likely have been obtained if it had been possible to also administer structured interviews, informant-reports or clinician ratings of DSM-5 traits. Thus, we recommend that ongoing research on the Norwegian PID-5 use informant or clinician reports of DSM-5 traits, which are currently available and free to use [5, 38].

## Conclusion

The results of this study suggest that the Norwegian PID-5-SF is an overall reliable, valid, and efficient measure of the DSM-5-AMPD trait system that can be considered largely equivalent to the original form of the PID-5.

## Additional file

Additional file 1: Beta weights from the regression analyses predicting PID-5 and PID-5-SF trait domains and facets from the BFI and PBQ-SF scales in the derivation sample. (DOCX 33 kb)

## Abbreviations

BFI: Big Five Inventory
DSM-5: Diagnostic and Statistical Manual of Mental Disorders 5^th^ edition
DSM-5 AMPD: Alternative DSM-5 Model for Personality Disorders
FFM: Five-factor model of personality
PBQ-SF: Personality Beliefs Questionnaire – Short Form
PID-5: Personality Inventory for DSM-5
PID-5-SF: Personality Inventory for DSM-5 Short Form
