# Supplementary material for: The Personality Inventory for DSM-5 Short Form (PID-5-SF): psychometric properties and association with big five traits and pathological beliefs in a Norwegian population
Source: BMC Psychol. 2016 Dec 7;4:61. doi: 10.1186/s40359-016-0169-5 (PMC5142430; doi:10.1186/s40359-016-0169-5)
Supplement: Additional file 1: — Beta weights from the regression analyses predicting PID-5 and PID-5-SF trait domains and facets from the BFI and PBQ-SF scales in the derivation sample. (DOCX 33 kb) [file 40359_2016_169_MOESM1_ESM.docx]

**Additional file 1**

**Table S1. Regression of each PID-5 domain scale on five BFI scales in the derivation sample**

|  | **BFI scales** | | | | | | | | | | | | | |  |  |  |  |  |
| --- | --- | --- | --- | --- | --- | --- | --- | --- | --- | --- | --- | --- | --- | --- | --- | --- | --- | --- | --- |
| **PID-5-SF domains** | **Neuroticism** | |  | **Extraversion** | |  | **Openness** | |  | **Agreeableness** | |  | **Conscientious-ness** | |  | **R^2^** | |  | **Profile agreement** |
|  | **O** | **D** |  | **O** | **D** |  | **O** | **D** |  | **O** | **D** |  | **O** | **D** |  | **O** | **D** |  |  |
| **Negative affect** | **.76** | **.73** |  | .05 | .06 |  | -.01 | -.02 |  | .02 | .03 |  | **-.08** | **-.08** |  | .60 | .54 |  | 1.00 |
| **Detachment** | **.14** | **.15** |  | **-.53** | **-.45** |  | .04 | .05 |  | **-.26** | **-.25** |  | **-.10** | **-.11** |  | .59 | .49 |  | .99 |
| **Psychoticism** | **.17** | **.11** |  | **-.19** | **-.19** |  | **.32** | **.31** |  | **-.25** | **-.24** |  | **-.23** | **-.17** |  | .40 | .31 |  | .99 |
| **Antagonism** | **-.15** | **-.14** |  | .08 | .06 |  | **.16** | **.12** |  | **-.52** | **-.52** |  | **-.11** | **-.09** |  | .29 | .28 |  | 1.00 |
| **Disinhibition** | **.18** | **.17** |  | .03 | .05 |  | **.08** | **.11** |  | **-.13** | **-.13** |  | **-.63** | **-.60** |  | .57 | .53 |  | 1.00 |

*Note*. *N* = 503. O = Original form; D = Derived short form. Standardized beta-coefficients are reported. Coefficients in bold are significant at *p* < 0.05, and the largest coefficient for each PID-5 domain is underlined.

**Table S2. Regression of each PID-5 scale on ten Personality Beliefs scales in the derivation sample**

|  | **PAR** | |  | **SCD** | |  | **ANT** | |  | **BDL** | |  | **HIS** | |  | **NAR** | |  | **AVD** | |  | **DPT** | |  | **OBS** | |  | **PAG** | |  | **R^2^** | | **Profile agreement** |
| --- | --- | --- | --- | --- | --- | --- | --- | --- | --- | --- | --- | --- | --- | --- | --- | --- | --- | --- | --- | --- | --- | --- | --- | --- | --- | --- | --- | --- | --- | --- | --- | --- | --- |
|  | **O** | **D** |  | **O** | **D** |  | **O** | **D** |  | **O** | **D** |  | **O** | **D** |  | **O** | **D** |  | **O** | **D** |  | **O** | **D** |  | **O** | **D** |  | **O** | **D** |  | **O** | **D** |  |
| **Negative affect** | .00 | .01 |  | **-.16** | **-.18** |  | -.06 | -.05 |  | **.31** | **.27** |  | .08 | .08 |  | -.03 | -.01 |  | **.23** | **.20** |  | **.30** | **.33** |  | **.10** | **.09** |  | -.01 | -.01 |  | .62 | .61 | .99 |
| **Anxiousness** | .08 | .08 |  | -.04 | -.05 |  | -.04 | -.04 |  | **.35** | **.34** |  | .02 | .02 |  | -.07 | -.02 |  | **.18** | **.16** |  | **.15** | .13 |  | **.15** | **.14** |  | -.05 | -.08 |  | .50 | .43 | .99 |
| **Emotional lability** | -.07 | -.06 |  | **-.13** | **-.10** |  | **-.14** | **-.12** |  | **.39** | **.35** |  | .10 | **.12** |  | .01 | .01 |  | **.25** | **.22** |  | .10 | .13 |  | -.01 | -.01 |  | .05 | .08 |  | .41 | .41 | .99 |
| **Perseveration** | -.06 | -.09 |  | **.15** | **.12** |  | .04 | .03 |  | **.19** | **.24** |  | .02 | .06 |  | **-.12** | **-.11** |  | -.01 | .07 |  | **.14** | .13 |  | **.26** | .09 |  | **.21** | **.21** |  | .44 | .38 | .84 |
| **Hostility** | -.07 | **-.15** |  | .09 | .04 |  | **.30** | **.20** |  | .03 | .09 |  | .03 | .04 |  | -.05 | -.06 |  | **.15** | **.20** |  | .10 | .14 |  | -.01 | .03 |  | **.22** | .10 |  | .40 | .28 | .82 |
| **Restricted affectivity** | **.18** | **.20** |  | **.37** | **.37** |  | **.15** | .10 |  | -.06 | -.05 |  | **-.12** | -.10 |  | **-.13** | **-.12** |  | -.04 | -.03 |  | .11 | .10 |  | .03 | .01 |  | **.14** | .11 |  | .36 | .31 | .99 |
| **Separation Insecurity** | -.03 | .00 |  | **-.29** | **-.29** |  | .04 | .04 |  | .00 | -.03 |  | **.09** | .07 |  | -.02 | .00 |  | **.15** | **.12** |  | **.56** | **.58** |  | .09 | **.09** |  | -.02 | -.02 |  | .53 | .50 | 1.00 |
| **Submissiveness** | -.10 | -.10 |  | -.04 | -.04 |  | **.15** | **.15** |  | -.09 | -.09 |  | **.15** | **.15** |  | **-.16** | **-.16** |  | **.19** | **.19** |  | **.34** | **.34** |  | **.12** | **.12** |  | -.10 | -.10 |  | .28 | .28 | 1.00 |
| **Detachment** | .05 | .04 |  | **.46** | **.44** |  | .01 | .04 |  | **.38** | **.42** |  | **-.17** | **-.18** |  | **-.13** | **-.12** |  | **.15** | **.11** |  | .02 | -.01 |  | .04 | .06 |  | .05 | .04 |  | .66 | .63 | .99 |
| **Anhedonia** | -.04 | -.04 |  | **.13** | .07 |  | -.01 | .06 |  | **.58** | **.57** |  | -.09 | -.08 |  | **-.13** | **-.14** |  | **.12** | .09 |  | .04 | .05 |  | -.03 | .03 |  | **.11** | .10 |  | .50 | .49 | .98 |
| **Depressivity** | -.04 | -.10 |  | .07 | .05 |  | .00 | .02 |  | **.70** | **.83** |  | -.02 | **-.11** |  | **-.21** | **-.14** |  | .10 | .08 |  | .00 | -.10 |  | .05 | .02 |  | .08 | .07 |  | .65 | .56 | .96 |
| **Intimacy avoidance** | .11 | .09 |  | **.54** | **.55** |  | .05 | .06 |  | .05 | .10 |  | **-.12** | **-.13** |  | **-.10** | **-.11** |  | .10 | .08 |  | .02 | -.01 |  | .03 | .02 |  | -.06 | -.09 |  | .40 | .39 | .99 |
| **Suspiciousness** | **.59** | **.48** |  | **.08** | **.10** |  | **.12** | **.11** |  | **.21** | **.30** |  | **-.12** | -.06 |  | -.07 | .02 |  | .01 | -.03 |  | .05 | .01 |  | -.04 | -.04 |  | -.01 | -.01 |  | .63 | .61 | .94 |
| **Withdrawal** | .07 | .03 |  | **.47** | **.43** |  | .00 | -.02 |  | **.29** | **.37** |  | **-.20** | **-.21** |  | **-.10** | -.06 |  | **.14** | .09 |  | -.01 | -.06 |  | **.09** | **.09** |  | .06 | .08 |  | .60 | .51 | .97 |
| **Psychoticism** | **.21** | **.20** |  | **.21** | **.21** |  | -.01 | .00 |  | **.23** | **.19** |  | -.03 | -.07 |  | -.04 | .01 |  | -.10 | -.09 |  | .10 | .06 |  | .01 | .00 |  | **.26** | **.26** |  | .47 | .40 | .98 |
| **Eccentricity** | **.21** | **.20** |  | **.23** | **.23** |  | -.09 | -.09 |  | **.26** | **.30** |  | -.02 | -.07 |  | -.06 | -.01 |  | -.10 | -.11 |  | .06 | .01 |  | .00 | .00 |  | **.27** | **.27** |  | .43 | .41 | .98 |
| **Perceptual dysregulation** | .10 | .04 |  | **.15** | .07 |  | .09 | **.18** |  | **.21** | .08 |  | .00 | **-.11** |  | **-.12** | .01 |  | -.02 | -.07 |  | **.15** | .11 |  | .01 | .00 |  | **.21** | **.23** |  | .41 | .21 | .67 |
| **Unusual beliefs** | **.24** | **.18** |  | **.12** | **.14** |  | .05 | .03 |  | .04 | -.01 |  | -.10 | -.01 |  | **.12** | .04 |  | -.13 | -.03 |  | .07 | .07 |  | .03 | .00 |  | **.18** | .11 |  | .27 | .17 | .77 |
| **Antagonism** | .00 | -.07 |  | **.12** | **.12** |  | **.39** | **.41** |  | -.05 | -.01 |  | **.18** | **.13** |  | **.19** | **.23** |  | -.05 | -.04 |  | -.11 | -.12 |  | -.05 | -.05 |  | .14 | **.16** |  | .49 | .49 | .98 |
| **Attention seeking** | -.03 | -.04 |  | .05 | .02 |  | **.12** | .07 |  | -.14 | **-.19** |  | **.56** | **.59** |  | **.15** | **.19** |  | **-.25** | **-.22** |  | .02 | .04 |  | -.05 | -.05 |  | .07 | -.02 |  | .37 | .34 | .98 |
| **Callousness** | .04 | -.02 |  | **.23** | **.23** |  | **.44** | **.42** |  | **.18** | .12 |  | **-.26** | **-.25** |  | **.12** | **.12** |  | **-.12** | -.07 |  | -.08 | -.02 |  | **-.13** | **-.15** |  | **.26** | **.20** |  | .55 | .42 | .98 |
| **Deceitfulness** | .00 | -.06 |  | **.11** | .10 |  | **.42** | **.44** |  | .05 | .13 |  | **.22** | **.20** |  | -.04 | .00 |  | .02 | -.01 |  | -.13 | **-.17** |  | **-.09** | -.08 |  | **.13** | **.13** |  | .40 | .38 | .97 |
| **Grandiosity** | .02 | -.07 |  | **.11** | **.11** |  | **.12** | **.17** |  | -.03 | .04 |  | -.05 | -.07 |  | **.56** | **.53** |  | **-.16** | -.08 |  | -.03 | -.05 |  | .00 | -.06 |  | .09 | **.12** |  | .45 | .44 | .96 |
| **Manipulativeness** | -.02 | -.05 |  | .07 | .08 |  | **.37** | **.37** |  | **-.23** | -.16 |  | **.22** | **.16** |  | .10 | .09 |  | -.03 | -.01 |  | -.07 | -.08 |  | .01 | .02 |  | **.12** | **.14** |  | .33 | .31 | .98 |
| **Disinhibition** | **-.16** | **-.12** |  | .09 | .08 |  | **.15** | **.15** |  | **.34** | **.30** |  | **.20** | **.22** |  | **-.21** | **-.19** |  | .04 | .02 |  | .03 | .05 |  | **-.18** | **-.17** |  | **.33** | **.30** |  | .40 | .37 | .99 |
| **Distractibility** | **-.15** | -.13 |  | **.10** | .07 |  | .03 | .01 |  | **.38** | **.35** |  | **.15** | **.13** |  | **-.21** | **-.17** |  | .03 | .01 |  | .07 | .08 |  | -.07 | -.03 |  | **.26** | **.23** |  | .35 | .30 | .99 |
| **Impulsivity** | -.05 | -.01 |  | -.01 | .01 |  | **.19** | **.18** |  | .08 | .10 |  | **.28** | **.26** |  | **-.18** | **-.18** |  | -.04 | -.05 |  | .01 | .04 |  | **-.21** | **-.20** |  | **.29** | **.25** |  | .20 | .21 | .99 |
| **Irresponsibility** | **-.20** | **-.16** |  | **.10** | **.12** |  | **.23** | **.22** |  | **.30** | **.24** |  | .10 | **.15** |  | -.10 | -.10 |  | .12 | .09 |  | -.04 | .00 |  | **-.23** | **-.21** |  | **.30** | **.23** |  | .34 | .29 | .97 |
| **Rigid perfectionism** | .07 | **.11** |  | **.13** | **.08** |  | -.06 | -.06 |  | -.05 | -.01 |  | **-.15** | **-.09** |  | .03 | -.01 |  | -.04 | -.05 |  | **.15** | .08 |  | **.75** | **.77** |  | -.02 | -.03 |  | .61 | .60 | .99 |
| **Risk taking** | **-.18** | **-.15** |  | .04 | .07 |  | **.20** | **.25** |  | .08 | .15 |  | **.21** | .10 |  | -.08 | -.05 |  | **-.35** | **-.24** |  | **-.22** | -.15 |  | .01 | .04 |  | **.34** | **.35** |  | .19 | .21 | .95 |

*Note*. *N* = 503. Standardized beta-coefficients are reported. Coefficients in bold are significant at *p* < .05 and the largest coefficient for each PID-5 scale is underlined. *R*^2^ indicates the degree to which all PBQ scales account for each PID-5 score (all *p* <.001). O = Original form; D = Derived short form. Personality Beliefs Questionnaire (PBQ) scales: Paranoid (PAR), Schizoid (SCD), Antisocial (ANT), Borderline (BDL), Histrionic (HIS), Narcissistic (NAR), Avoidant (AVD), Dependent (DPT), Obsessive-Compulsive (OBS), and Passive-Aggressive (PAG).
